# *Drosophila* Plc21C is involved in calcium wave propagation during egg activation

**DOI:** 10.17912/micropub.biology.000235

**Published:** 2020-04-01

**Authors:** Qinan Hu, Adriana N. Vélez-Avilés, Mariana F. Wolfner

**Affiliations:** 1 Department of Molecular Biology and Genetics, Cornell University, Ithaca, NY 14853, USA; 2 University of Puerto Rico-Río Piedras, Río Piedras, PR

**Figure 1:**
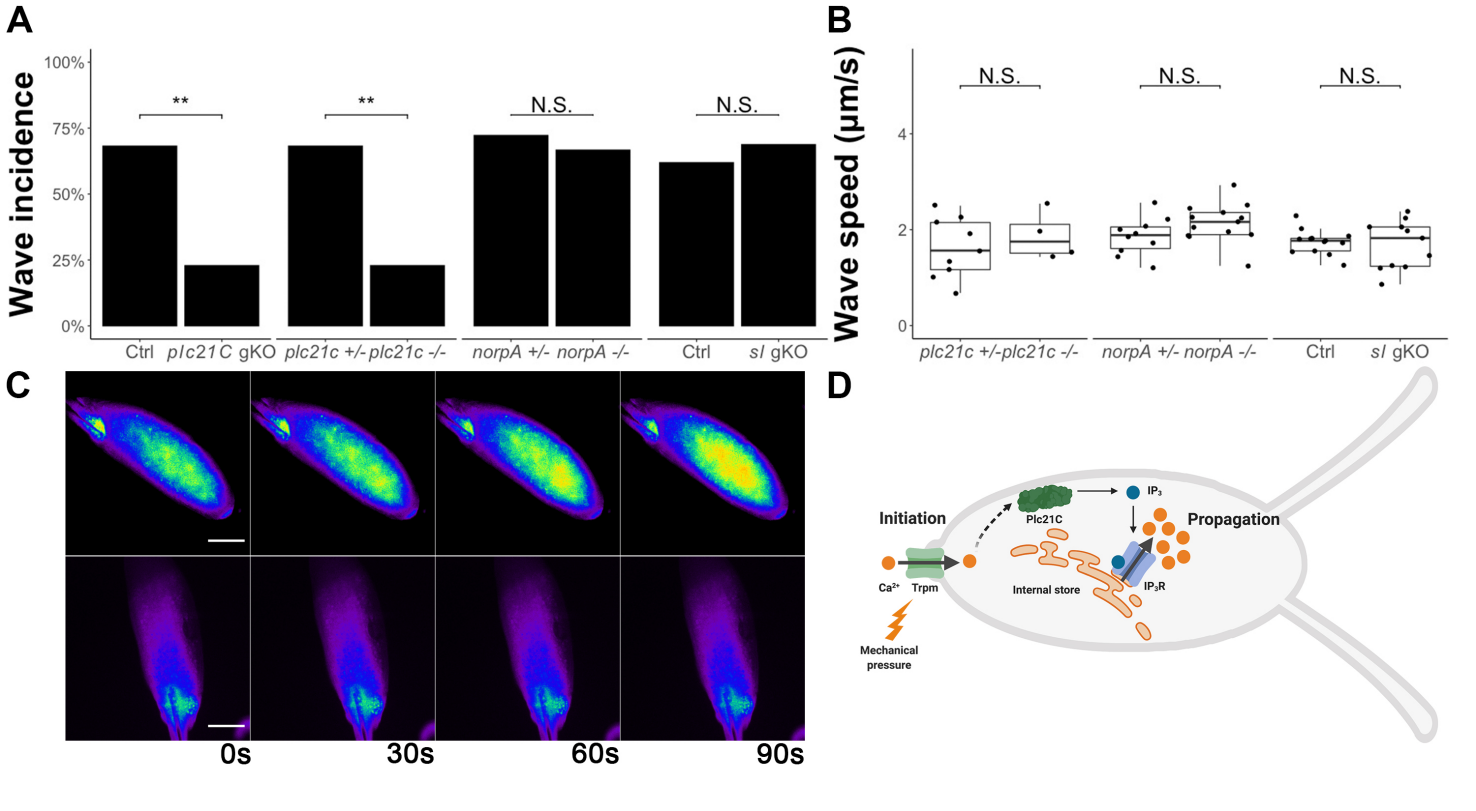
***plc21C*,but not *norpA* or *sl*, is necessary but not sufficient for calcium wave incidence *in vitro*. (A)** The incidence of calcium waves in oocytes from (left to right):*plc21C* germline-knockout females (*nos-Cas9; gRNA-plc21C*, n=16, p=0.007);*plc21C^1^* null mutant females (n=35, p=0.0018); *norpA^36^* null mutant females(n=18, p=1); *sl* germline knockout mutant(*nos-Cas9; gRNA-sl*, n=16, p=0.93) compared with controls (*nos-Cas9*, n=21; *plc21C^1^/*+, n=22; *norpA^36^*/+, n=15; *nos-Cas9*, n=21, respectively) during *in vitro* egg activation.**(B)** The propagation speed of calcium waves in (left to right): the few oocytes from *plc21C^1^* null mutant females that had calcium waves (n=4, 1.87±0.51 μm/s, p=0.47); oocytes from *norpA^36^* null mutant females (n=10, 2.13±0.41 μm/s); oocytes from *sl* germline knockout females (1.68±0.50 μm/s, n=11), p=0.72, all compared with their controls (*plc21C^1^*/+, 1.62±0.62 μm/s, n=9; *norpA^36^*/+, 1.85±0.39 μm/s, n=13; *nos-Cas9*, 1.74±0.26 μm/s, n=13, respectively) **(C)** Representative images of calcium waves, or lack thereof, in control (top, *plc21C^1^*/+, n=15/22) and *plc21C^1^* null mutant (bottom, n=8/35). A pseudocolor look-up table was applied to make the signals easier to see. **(D)** Proposed model of the initiation and propagation of calcium waves during *Drosophila* egg activation. Image made using Biorender.com. All scale bars = 100 μM. **: p<0.01, N.S.: not significant, gKO: germline-specific knockout.

## Description

Mature oocytes arrest in meiosis by the end of oogenesis and need to be activated in order to proceed to embryonic development. This egg activation process encompasses a series of events that transition the oocyte to a developing embryo, including meiosis resumption, maternal protein translation/modification, maternal mRNA processing, and cytoskeleton and eggshell changes (reviewed in Avilés-Pagán and Orr-Weaver, 2018; Horner and Wolfner, 2008a; Kashir *et al.*, 2014; Krauchunas and Wolfner, 2013). The triggers of egg activation vary across species, from mechanical pressure in arthropods to the fertilizing sperm in nematodes, echinoderms and vertebrates (reviewed in Horner and Wolfner, 2008a). Despite differences in trigger, a rise of intracellular calcium levels preceding downstream events is found in most species studied to date (reviewed in Swann and Lai, 2016). In *Drosophila* egg activation, the intracellular calcium rise is triggered by mechanical cues, which can be pressure exerted by the oviduct or from oocyte swelling *in vivo* (Heifetz *et al.*, 2001) or *in vitro* due to osmotic pressure from a hypotonic buffer (Horner and Wolfner, 2008b; Page and Orr-Weaver, 1997). This calcium rise takes the form of a transient calcium wave starting from the pole(s) and traversing the oocyte (Kaneuchi *et al.*, 2015; York-Andersen *et al.*, 2015). This calcium wave is initiated by influx of environmental calcium through Trpm channels in response to the mechanical trigger (Hu and Wolfner, 2019). Further propagation of the calcium wave requires release of internal calcium from stores through the inositol 1,4,5-trisphosphate (IP_3_) receptor (IP_3_R) calcium channel (Kaneuchi *et al.*, 2015). It remains unclear how the initiation of calcium waves triggers the activation of IP_3_R during this process.

Phospholipase Cs (PLCs) are membrane-associated enzymes that mediate the cleavage of phospholipids, specifically the cleavage of phosphatidylinositol 4,5-bisphosphate (PIP_2_) to produce diacylglycerol (DAG) and IP_3_. This reaction is involved in multiple signal transduction pathways (reviewed in Kadamur and Ross, 2013). In mammalian egg activation, a sperm-delivered PLC (PLCζ) is responsible for activating IP_3_R to start the initial calcium rise (Saunders *et al.*, 2002).

Because the propagation of calcium waves in *Drosophila* egg activation also requires IP_3_R, we hypothesized that this is also mediated by PLC and the IP_3_ pathway. The *Drosophila* genome encodes three PLCs: No receptor potential A(*norpA*), Small wing(*sl*) and Phospholipase C at 21C(*plc21C*). According to the RNAseq database, all three are expressed in *Drosophila* ovaries (Leader *et al.*, 2017). It is possible that one or more of the three PLCs is involved in transducing the initial calcium influx signal to the IP_3_ pathway to allow the calcium wave to propagate. Some PLCs can directly bind to Ca^2+^ and get activated in response to calcium signals (reviewed in Katan, 1998). All three *Drosophila* PLCs contain EF hand domains, which can potentially bind Ca^2+^ and directly transduce the ionic signal to downstream pathways (Lewit-Bentley and Réty, 2000).

To determine the role of phospholipase C (PLC) in calcium wave propagation during *Drosophila* egg activation, we screened each of the PLCs. We started by examining the role of Plc21C. We examined the calcium wave phenotype in oocytes from germline-specific CRISPR/Cas9 *plc21C* knockout females. These females were offspring from *nos-Cas9; matα-GAL4-VP16; UAS-GCaMP3* crossed to *gRNA-plc21C* (see Methods). We observed a significant decrease in calcium wave incidence in oocytes from *plc21C* germline knockout females compared to controls (**Fig.1A**). To confirm these results, we isolated a null allele of *plc21C* (*plc21C^1^*) from the offspring of the germline-knockout females (see Methods). This mutation is homozygous viable. We crossed it into the *nos-GCaMP6m* transgenic background (Hu and Wolfner, 2019) to allow us to visualize calcium dynamics in the germline of *plc21C^1^* females. We examined oocytes dissected from *plc21C^1^* homozygous females during *in vitro* egg activation and again found a significant decrease in calcium wave incidence compared to heterozygous controls (**Fig.1A and C**). In the few homozygous *plc21C^1^* oocytes that did show calcium waves, we did not observe a significant difference in calcium wave propagation speed compared to controls (**Fig.1B**). Taken together, our data show that calcium waves during *Drosophila* egg activation requirePlc21C.

Since there were still calcium waves in a minority of *plc21C^1^* mutant oocytes, we suspected that Plc21C might function redundantly with other molecule(s). We thus examined the role of the two other PLCs encoded by the *Drosophila* genome, NorpA and Sl. *norpA* has an available, viable, null allele *norpA^36^* (Riesgo-Escovar *et al.*, 1995). We crossed it into it the *nos-GCaMP3-attP2* transgenic background (Kaneuchi *et al.*, 2015) to visualize calcium dynamics in the germline. We isolated mature oocytes from *norpA^36^; nos-GCaMP3-attP2* females and imaged them during *in vitro* egg activation. We observed that calcium wave incidence and propagation speed did not differ between the oocytes of homozygous *norpA^36^* mutants and heterozygous controls (**Fig.1A-B**). Next, we examined calcium waves in *sl* germline knockout oocytes during *in vitro* activation. Mutant oocytes did not differ from control oocytes in calcium wave incidence or propagation speed (**Fig.1A-B**). We also isolated a null allele of *sl* (*sl^12^*) from the offspring of *sl* germline-knockout females (see Methods) and attempted to visualize calcium waves in oocytes from *sl^12^* females. However, *sl^12^* appeared to have combinatorial lethality with the *nos-GCaMP6m* transgene, as we were unable to isolate homozygous *sl^12^; nos-GCaMP6m* flies. The fluorescence signal strength of heterozygous *nos-GCaMP6m* was too low for us to visualize calcium waves. Although oocytes from germline specific *sl* knockout females displayed normal calcium wave incidence and propagation speed, it is possible that this knockout did not efficiently cause biallelic null mutations in most oocytes to reveal the function of Sl. Thus, we were unable to determine a requirement of *sl* for calcium wave propagation.

The presence of calcium waves in a minority of *plc21C* null oocytes suggests that Plc21C functions redundantly with other PLCs such as Sl or with other calcium signal relaying mechanisms to facilitate calcium wave propagation. These redundant mechanisms require further investigation. It also remains to be elucidated how Plc21C is activated by the initial calcium influx, whether through direct binding of Ca^2+^ to Plc21C or through other signal relaying molecules. Finally, we note that lack of Plc21C activity did not lead to the presence of initiated but only partially-propagated calcium waves, as was seen for IP3R knockdowns (Kaneuchi *et al.*, 2015). The complete absence of calcium waves seen in most *plc21C* null oocytes suggests that Plc21C activity is needed at the earliest stages of (or to initiate) wave propagation in response to the Trpm-mediated calcium influx.

In wing imaginal discs, Plc21C is required for the intercellular calcium waves that regulate wing development via the IP_3_ pathway (Brodskiy *et al.*, 2019). Thus, Plc21C and the IP_3_ pathway mediate both intracellular calcium waves during egg activation and intercellular calcium waves during tissue development.

This study identified the connection between calcium wave initiation and propagation during *Drosophila* egg activation. Based on this and our previous studies (Hu and Wolfner, 2019; Kaneuchi *et al.*, 2015), we propose the following model for the mechanism of the calcium wave during *Drosophila* egg activation: mechanical pressure activates Trpm channels located on the plasma membrane of mature oocytes, allowing influx of external calcium. These Ca^2+^ ions then directly or indirectly activate Plc21C (and possibly other signal-relaying molecules), which catalyzes the reaction producing IP_3_. IP_3_ then binds to and activates its receptor to release calcium from internal stores, facilitating propagation of the calcium wave (**Fig.1D**). Our demonstration of the use of PLC to relay egg activation triggering signals to intracellular calcium rises reveals an important conservation in egg activation mechanisms between *Drosophila* and mammals.

## Methods

**Fly strains and maintenance**

All *Drosophila* strains and crosses were maintained or performed on standard yeast-glucose-agar media at 25C° on a 12/12 light/dark cycle. The *nos-Cas9; matα-GAL4-VP16; UAS-GCaMP3* transgenic line was made by crossing *yw, nos-Cas9* into a previously described *matα-GAL4-VP16; UAS-GCaMP3* transgenic line (Kaneuchi *et al.*, 2015). The *nos-GCamP3* and *nos-GCaMP6m* transgenic lines were as previously described (Hu and Wolfner, 2019; Kaneuchi *et al.*, 2015). *norpA^36^* (9048) and *yw, nos-Cas9* (54591) fly lines were obtained from the Bloomington *Drosophila* Stock Center.

**DNA constructs and transgenic flies**

Calcium waves were visualized by expressing GCaMP calcium sensors in the female germline using *matα4-GAL4-VP16; UASp-GCaMP3* or *nos-GCaMP6m* as previously described (Hu and Wolfner, 2019; Kaneuchi *et al.*, 2015). To generate CRISPR/Cas9 knockouts of *plc21C* and *sl*, we followed the previously described germline specific CRISPR/Cas9 genome editing protocol (Hu and Wolfner, 2019; Poe *et al.*, 2018). The offspring of the germline knockout females were isolated and sequenced to establish stable lines of *plc21C* and *sl* null mutants. The following gRNA target sequences were used:

*gRNA-plc21C*: CTACATCTCCACCGCCAGCG; CTTCTGGAACGGACGCACCG

*gRNA-sl*: ACCATTGGTATGCTGGAGCG; CTCCAGTGAATCCTCCTGCG

These gRNA expression constructs were injected by Rainbow Transgenic Flies, Inc. into *yw, nos-phiC31; PBac{attP-9A}* embryos. Flies carrying correct insertions were isolated to establish gRNA expression transgenic lines. To generate whole fly knockout strains for *plc21C* and *sl*, we crossed germline knockout females to males carrying balancer chromosomes. The F1 progeny were single-pair mated with balancer flies. Once the crosses began producing offspring the parent containing a putative PLC mutation was individually genotyped with PCR. Primers flanking the gRNA targeting sites were used in PCR to detect deletions. Primer sequences are as follows: *plc21C-*F: TCGGATACCAACCAGGACTATG, *plc21C*-R: TATCTCGGGCACGAACGTATAG; *sl*-F: CGGATGAGAACTGGATTCGATAG, *sl*-R: GTGCAGTATGACAAAGCACTTG. The F2 progeny of crosses from the confirmed-mutant F1s were brother-sister mated to establish stable mutant lines. *plc21C^1^* carries a ~19kb deletion from exon 1 to exon 8, covering the majority the gene. *sl^12 ^*carries a 44 bp deletion in exon 1 that leads to a frameshift and premature stop codon.

***In vitro* egg activation assay and imaging**

Oocytes were dissected from the indicated female flies fattened on yeast and were induced to activate *in vitro* following methods as previously described (Hu and Wolfner, 2019; Kaneuchi *et al.*, 2015). Before imaging, oocytes were placed in a drop of Isolation Buffer (IB) (Page and Orr-Weaver, 1997) in a glass-bottomed Petri dish. IB was then replaced by modified Robb’s buffer (RB) (Hu and Wolfner, 2019) to induce egg activation at the start of imaging. Time-lapse images were taken at every 1s for 20 min after the addition of RB, using Zeiss Elyra Super Resolution Microscope with a 10X lens and Zen software. The detection wavelength was set to 493-556 nm, for the GCaMP signal.

**Statistics**

Pearson’s *χ^2^* test was used to compare the incidence of calcium waves. Student’s *t* test was used to compare the propagation speeds of the calcium waves.
