# Corrigendum: *Drosophila* Plc21C is involved in calcium wave propagation during egg activation

**DOI:** 10.17912/micropub.biology.000240

**Published:** 2020-04-11

## Description

For Hu, Q; Vélez-Avilés, AN; Wolfner, MF (2020). *Drosophila* Plc21C is involved in calcium wave propagation during egg activation. microPublication Biology. 10.17912/micropub.biology.000235

Authors requested to correct an omission in their funding acknowledgement:

“NIH grant R21-HD088744 (to MFW). NSF Grant 1428922 funding (for the shared Zeiss Elyra Super Resolution Microscope).”

The authors correct the funding report to read:

“NIH grant R21-HD088744 (to MFW). NSF Grant 1428922 funding (for the shared Zeiss Elyra Super Resolution Microscope). For ANVA: partial support from Cornell’s Department of Molecular Biology and Genetics and its NSF-funded REU program (funded on NSF REU DBI-1659534), and partial support from the Society of Developmental Biology’s Choose Development! Program, which is funded by NSF (IOS-1239422; Broadening participation of underrepresented groups in Developmental Biology and REU DBI-1156528).”

This omission has been corrected online.

